# A Control Systems Approach to Quantify Wall Shear Stress Normalization by Flow-Mediated Dilation in the Brachial Artery

**DOI:** 10.1371/journal.pone.0115977

**Published:** 2015-02-18

**Authors:** Frank C. G. van Bussel, Bas C. T. van Bussel, Arnold P. G. Hoeks, Jos Op 't Roodt, Ronald M. A. Henry, Isabel Ferreira, Floris H. M. Vanmolkot, Casper G. Schalkwijk, Coen D. A. Stehouwer, Koen D. Reesink

**Affiliations:** 1 Department of Biomedical Engineering, Maastricht University Medical Centre+, Maastricht, The Netherlands; 2 Department of Radiology, Maastricht University Medical Centre+, Maastricht, The Netherlands; 3 Department of Internal Medicine, Maastricht University Medical Centre+, Maastricht, The Netherlands; 4 Department of Clinical Epidemiology and Medical Technology Assessment, Maastricht University Medical Centre+, Maastricht, The Netherlands; 5 School for Nutrition, Toxicology and Metabolism (NUTRIM), Maastricht University Medical Centre+, Maastricht, The Netherlands; 6 School for Cardiovascular Diseases (CARIM), Maastricht University Medical Centre+, Maastricht, The Netherlands; 7 School for Mental Health and Neuroscience (MHeNS), Maastricht University Medical Centre+, Maastricht, The Netherlands; University of Messina, ITALY

## Abstract

Flow-mediated dilation is aimed at normalization of local wall shear stress under varying blood flow conditions. Blood flow velocity and vessel diameter are continuous and opposing influences that modulate wall shear stress. We derived an index FMD_v_ to quantify wall shear stress normalization performance by flow-mediated dilation in the brachial artery. In 22 fasting presumed healthy men, we first assessed intra- and inter-session reproducibilities of two indices pFMD_v_ and mFMD_v_, which consider the relative peak and relative mean hyperemic change in flow velocity, respectively. Second, utilizing oral glucose loading, we evaluated the tracking performance of both FMD_v_ indices, in comparison with existing indices [i.e., the relative peak diameter increase (%FMD), the peak to baseline diameter ratio (D_peak_/D_base_), and the relative peak diameter increase normalized to the full area under the curve of blood flow velocity with hyperemia (FMD/shear_AUC_) or with area integrated to peak hyperemia (FMD/shear_AUC_peak_)]. Inter-session and intra-session reproducibilities for pFMD_v_, mFMD_v_ and %FMD were comparable (intra-class correlation coefficients within 0.521–0.677 range). Both pFMD_v_ and mFMD_v_ showed more clearly a reduction after glucose loading (reduction of ~45%, p≤0.001) than the other indices (% given are relative reductions): %FMD (~11%, p≥0.074); D_peak_/D_base_ (~11%, p≥0.074); FMD/shear_AUC_peak_ (~20%, p≥0.016) and FMD/shear_AUC_ (~38%, p≤0.038). Further analysis indicated that wall shear stress normalization under normal (fasting) conditions is already far from ideal (FMD_v_ << 1), which (therefore) does not materially change with glucose loading. Our approach might be useful in intervention studies to detect intrinsic changes in shear stress normalization performance in conduit arteries.

## Introduction

Dilation of vascular beds in response to an increased blood volume flow is an essential mechanism in (early) vascular development and in adult circulatory adaptation [1–3]. Current insight is that shear stress, sensed and transduced by the glycocalyx-endothelium apparatus, plays a major role in the acute and long-term adaptation of vascular diameter [2,3]. Dysfunction of the endothelial mechano-transduction is commonly assessed non-invasively using ultrasound echography by measuring brachial artery dilation as elicited by a hyperemic flow-stimulus [4–6] and commonly quantified as the relative peak increase in brachial artery diameter (%FMD) [7–11]. A low %FMD is associated with (cardio)vascular events [12,13] and is considered a subclinical marker of vascular dysfunction as may occur in hypertension, diabetes, and hypercholesterolemia [5,11].

Despite improvements in the measurement of brachial artery %FMD [6–8,14] its measurement is associated with poor reproducibility [14–18]. This might be explained by the fact that stimulus magnitude is not accounted for [19–26]. The commonly used hyperemic flow-stimulus is subject to health status [10,11] and may by itself be modulated by the interventions studied [27,28]. Although several ways to incorporate stimulus magnitude have been proposed and advocated [9,11,29,30], how to account for stimulus magnitude remains subject of ongoing debate [31].

Since mean wall shear stress is directly related to the ratio of mean blood flow velocity and vessel diameter [9,32,33], these two variables have opposing influences on local wall shear stress. We hypothesized that the continuous balance between these influences could be used to more accurately quantify the stimulus-response relation in flow-mediated dilation measurements.

What follows is a step-by-step derivation of an index that fully captures the stimulus-response relationship.

Time averaged blood flow through the brachial artery can be approximated by an incompressible liquid with constant viscosity moving within a straight, long tube, thus having a parabolic velocity distribution. These presumptions are valid for flow-mediated dilation assessment, where one considers a time-scale of many times the cardiac cycle and consequently measure time-average blood flow velocity and vessel diameter. According to Poiseuille's law, the flowing blood exerts a frictional stress on the inner lining of the vessel, defined as wall shear stress (τ_w_). For a given blood viscosity (η) and a presumed parabolic velocity profile, brachial artery τ_w_ is a function of the local centre-stream blood flow velocity (v) and diameter (D), according to [3]:

$${\text{τ}}_{\text{w}}=\frac{\text{8ηv}}{\text{D}}$$ (eq.1)

In a first order Taylor approximation, any change in τ_w_ can be expressed as a difference equation with respect to velocity and diameter:

$${\text{Δτ}}_{\text{w}}=\frac{\text{8η}}{{\text{D}}_{\text{base}}}\text{Δv}-\frac{{\text{8ηv}}_{\text{base}}}{{\text{D}}_{\text{base}}^{\text{2}}}\text{ΔD}$$ (eq.2)

The first term on the right hand side of equation 2 reflects the change in wall shear stress evoked by a change (∆v = v-v_base_) in blood flow velocity, while the second term reflects the opposite change in wall shear stress evoked by a change (∆D = D-D_base_) in diameter. The efficacy with which the diameter change counteracts any velocity induced change in wall shear stress is then given by the ratio of the corresponding terms:

FMDv=8ηvbase ΔDDbase28ηΔvDbase=ΔDDbaseΔvvbase (eq.3)

From the above equation it follows that this velocity normalized index FMD_v_ has a lowest value of 0 in the absence of a diameter response (∆D = 0). In contrast, increasing values of FMD_v_ indicate more effective restoration of mean wall shear stress to baseline, despite the increased volume flow through the artery.

**Figure 1:**
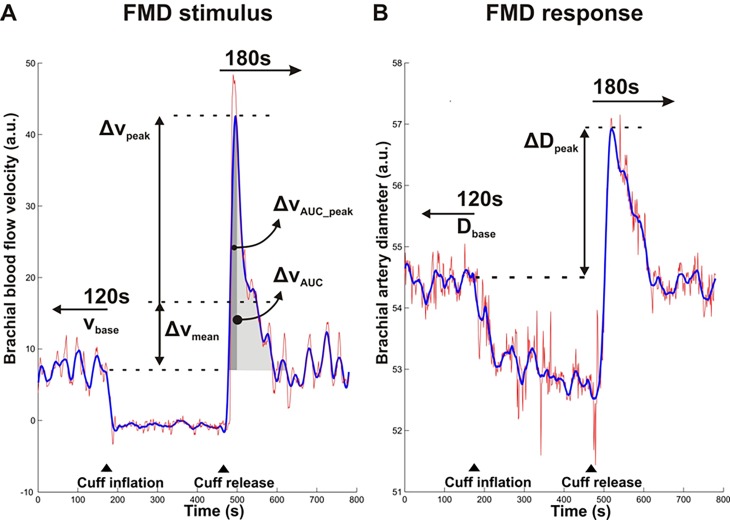
Definitions of baseline and hyperemic brachial artery blood flow velocity and diameter parameters for the assessment of flow-mediated dilation. Blood flow velocity (A) and diameter curves (B) obtained by beat-to-beat analysis of Duplex video images. Raw data curves (red) were smoothed (blue) to reduce noise, particularly in the peak change estimates. Baseline blood flow velocity (v_base_) and diameter (D_base_) were determined over the 120 seconds prior to cuff-inflation. The peak change in blood flow velocity (Δv_peak_) was determined as the maximum velocity reached within 180 seconds after cuff-release while the mean change in blood flow velocity (Δv_mean_) was determined as the average velocity over the 180 seconds after cuff-release, both minus the baseline blood flow velocity v_base_ (A). The peak change in diameter (ΔD_peak_) was determined as the maximum diameter reached within 180 seconds after cuff-release minus the baseline diameter D_base_ (B). Indicated also are the full area under the curve of the hyperemic velocity curve above baseline level (Δv_AUC_) and the area integrated till peak-time of velocity curve (Δv_AUC_peak_). ▲indicates the timing of rapid cuff inflation and release.

Equation 3 is derived for a steady state situation where the hyperemic state is compared with the baseline. The selection of appropriate parameters for the transient case is less obvious and depends on the anticipated reproducibility. Since the change in mean diameter is limited and might be more susceptible to baseline drift, we, as is commonly done, also opted for the peak change in diameter. On the other hand, the velocity exhibits a marked change with hyperemia, permitting the selection of either the peak or mean change in velocity, resulting in the following specific indices (see Fig. 1 for definition of parameters):

pFMDv=(Dpeak – Dbase)Dbase(vpeak – vbase)vbase (eq.4)

mFMDv=(Dpeak – Dbase)Dbase(vmean – vbase)vbase (eq.5)

In the present study, we evaluated (1) the reproducibility and (2) the tracking performance of the pFMD_v_ and mFMD_v_ indices in comparison with existing FMD indices [8,9,14,34–36] in 22 presumed healthy male volunteers undergoing a standard oral glucose tolerance test, at two occasions. Oral glucose loading has been consistently shown to reduce brachial artery %FMD in healthy individuals [19–24].

## Methods

### Study population

Twenty-two presumed healthy men (age range 18–46 yrs) participated. None had a history of diabetes, myocardial infarction or cardiovascular disease, or used medication. Height and weight were measured and body mass index (BMI, in kg/m^2^) was calculated as the ratio of weight over height squared. Smoking status was determined by a questionnaire and classified as none, occasionally, or daily smoking. Pre-inclusion, fasting glucose was determined (OneTouch Ultrasmart, LifeScan, California, USA); each participant complied with the study limit, set at ≤ 6.1 mmol/l. Blood pressure was measured with an automated oscillometric device with the cuff positioned around the left upper arm (Omron 705 IT, Omron Healthcare Europe B.V., Hoofddorp, the Netherlands).

The study was approved by the medical ethics committee of the Maastricht University Medical Centre (MUMC) and all participants gave written informed consent prior to enrolment.

### Study protocol

Participants were asked to refrain from exercise, smoking, vitamin supplement use, and consumption of caffeine, alcohol, liquid (except water until 3 hours prior the start of measurements) or food twelve hours prior to the start of measurements. These were performed in the morning, with participants in supine position, in a quiet, climate-controlled room (22–23°C) with dimmed lights, after a 15 minute period of acclimatization. A cannula was inserted into the left antecubital vein for blood sampling.

**Figure 2:**
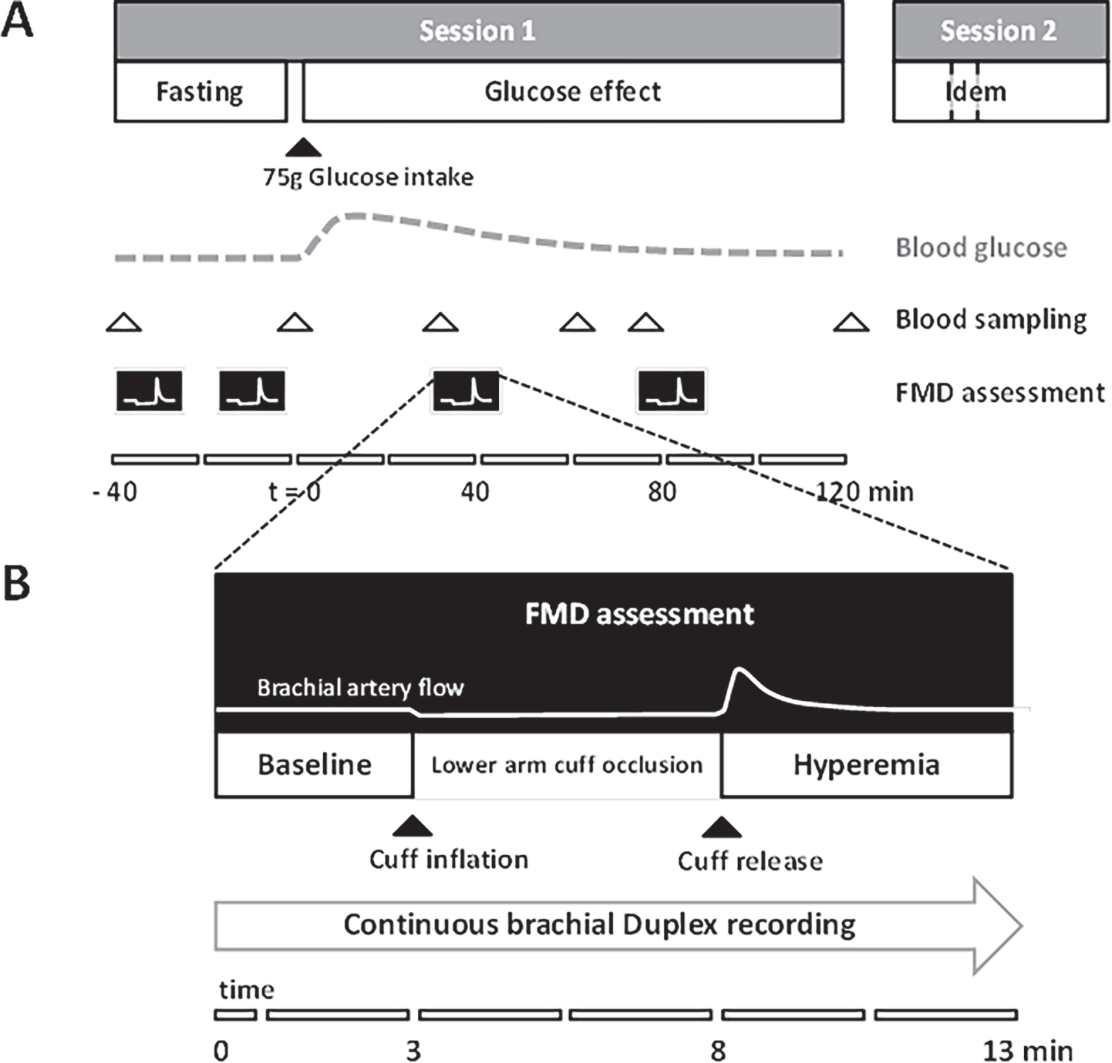
Detailed timelines of (A) the study protocol and (B) the flow-mediated dilation assessment. (A) The effect on brachial artery flow-mediated dilation after glucose loading was assessed repeatedly, sessions 1 and 2 about one week apart. (B) A 5-minute lower arm cuff-occlusion was used to elicit a hyperemic response to measure flow-mediated dilation from a continuous brachial Duplex ultrasound recording (B-mode and pulsed Doppler). Note the stimulus-response type of assessments for both (A) and (B) and the correspondingly different timescales.

Participants underwent twice a set of measurements (spaced 8.5 ± 3.4 days apart) including an oral glucose tolerance test (OGTT; 82.5g dextrose monohydrate in 250 ml water plus additional 50 ml water) at the University's Research Unit (Fig. 2). In each session, four flow-mediated dilation assessments were performed: two repeated measurements spaced ten minutes apart prior to glucose intake and two follow-up measurements after 30 and 75 minutes, respectively (Fig. 2).

### Flow-mediated dilation measurement

The right shoulder and arm of the subjects were positioned on soft supports for optimal comfort and stability, to avoid muscle tension build-up and subsequent movement. Upper arm and wrist were rested on soft supports to avoid positioning artifacts due to cuff-inflation. A well-accessible segment of the right brachial artery was interrogated using a 7.5 MHz, 40 mm linear-array ultrasound system (MyLab 70, Esaote, Maastricht, the Netherlands) in Duplex-mode to record simultaneously artery diameter and center-line blood flow sonogram. Emitted radiofrequency was set for highest resolution. The transducer position was fixed using a stereotactic probe holder, which provided all degrees of freedom to orient the probe at insonation angles perpendicular to the vessel long axis. The electronically steered pulsed-Doppler beam was set at an angle of 65–70 degrees with respect to the vessel long axis/flow direction. The length of the pulsed-Doppler sample volume was set at approximately half the vessel diameter to ensure consistent registration of the center-line velocity despite possible movements over the recording period. Readjustment of the sample volume was avoided as much as possible because of the associated Doppler artifacts. A sphygmomanometer cuff (Hokanson TD 312, D.E. Hokanson, Inc., Bellevue, USA) was positioned on the right lower arm and inflated to 200 mmHg for 5 minutes to elicit upon release the hyperemic response required for brachial artery flow-mediated dilation measurement (Fig. 1B), as recommended [6–8]. The cuff position was marked to ensure repeatable cuff repositioning within each session. For each flow-mediated dilation measurement, duplex images were recorded continuously on DVD over a total of 13 minutes: 3 minutes baseline (before rapid cuff-inflation), 5 minutes during lower arm cuff-inflation, and 5 minutes during hyperemia following rapid cuff-release (Fig. 2B). A lead II ECG was recorded concurrently with the Doppler velocity signal to facilitate beat-by-beat measurements of brachial artery diameter and blood flow velocity in off-line video analysis.

### Duplex video analysis

Recorded duplex video images were analyzed off-line using proprietary wall detection and Doppler velocity tracing software (Department of Biomedical Engineering, University of Maastricht, the Netherlands) implemented in Matlab (The Mathworks, Natick, MA). (A) Processing started with manual identification of the region of interest (ROI) within the B-mode image, containing a straight section of the artery. (B) Subsequently, the approximate position of the vessel wall-lumen interface was manually identified for both the anterior and posterior wall at the left and right side of the ROI. The lines through these points served as reference for the search region (+/- 1.5 mm) for the wall-lumen interface in subsequent images. (C) The video frame coinciding with an ECG R-wave (i.e. end-diastolic) was automatically identified, utilizing the intensity coding of the ECG-waveform in the echo image. (D) For those images, first the search region was explored for the local echo maximum, reflecting the media-adventitia interface. The instantaneous wall position was identified starting from within the lumen as the first level-crossing (65%) relative to the adventitia level (100%), and provided an updated reference for the search region. The distance between the anterior and posterior wall positions averaged over the ROI was the estimate for the instantaneous diameter. (E) The instantaneous mean blood flow velocity was based on the spectral mean of the intensity distribution of the sonogram for each time instance, taking into account positive as well as negative flow-velocities (that typically occur in early diastole under baseline conditions). Its average over the sonogram, covering typically 3–5 heart cycles, was used for further processing. (F) The velocity and diameter samples were resampled at 1Hz and low-pass filtered by a third order zero phase Savitsky-Golay filter with a width of 17 and 35 seconds, respectively, resulting in recordings as illustrated in Fig. 1A (velocity) and 1B (diameter), respectively. (G) Subsequently, the start and end of cuff-occlusion were manually identified, facilitating automatic extraction of the following parameters: (i) the baseline blood flow velocity (v_base_) and the baseline diameter (D_base_) are medians over the 120 seconds prior to cuff-inflation; (ii) the absolute peak change in blood flow velocity (Δv_peak_) and the absolute peak change in diameter (ΔD_peak_) are the peaks of the velocity and diameter curves within the first 180 seconds after cuff-release relative to their baseline values (Fig. 1A and B); (iii) the mean change in velocity (Δv_mean_) is the average of the curve for the same period minus the baseline value, and (iv) the *relative* peak change in blood flow velocity, the *relative* mean change in blood flow velocity and *relative* peak change in diameter are calculated as Δv_peak_/v_base_, Δv_mean_/v_base_ and ΔD_peak_/D_base_, respectively. FMD_v_ indices were calculated as given in the introduction (Eq. 4 and Eq. 5).

The automated determination of abovementioned values was verified by visual inspection of the recordings (as shown in Fig. 1A and B), since (movement) artifacts and baseline drift may render automatic quantification invalid [6–8]. Verification was performed blinded for the session and time-point of the flow-mediated dilation measurement, in random order, and prior to further data-analysis. As a result of this blinded procedure, 3% of the total 176 recordings were fully excluded. Of the included measurements, 10% of baseline diameter, 3% of peak diameter and 44% of baseline blood flow velocity values were manually corrected.

To compare the FMD_v_ approach with existing indices that normalize the dilatory response to stimulus strength we calculated the relative peak diameter increase-to-hyperemic area under the curve (AUC) of blood flow velocity, obtained by integration (i) from cuff-release till peak velocity [9] and (ii) over de full response (till 180 seconds) [36]: (ΔD_peak_/D_base_)/Δv_AUC_peak_ and (ΔD_peak_/D_base_)/Δv_AUC_, respectively (see also Fig. 1 for definitions). The corresponding indices originally normalize to shear rate and hence need to take into account blood viscosity and a parabolic velocity profile to estimate shear rate from mean or center-line velocity. However, our focus is on tracking ability within individuals and short-term, i.e. relative changes with glucose-loading, which makes these factors irrelevant for our comparative analysis.

### Blood sample processing

All blood samples were centrifuged at 3000 rpm for 15 minutes and plasma was stored in -80°C freezers until further analysis. The glucose concentration was determined by standardized glucose oxidase method (Synchron LX20 pro, Beckman Coulter Inc. CA, USA), with intra- and inter-assay coefficients of variation of 2.0% and 3.0%, respectively.

### Statistical analysis

First, we evaluated the reproducibility of the pFMD_v_ and mFMD_v_ indices in comparison with %FMD. Data are reported as means ± SD. Test-retest agreement estimates were calculated on data of the first and second flow-mediated dilation assessments of both sessions (i.e. all fasting flow-mediated dilation assessments) [37]. Intra-session and inter-session intra-class correlation coefficients (ICC) and coefficients of variation (CV = 100%·SD/mean) were calculated using ANOVA [37,38]. Bland-Altman plots of the differences between the data of the first and second session (inter-session) versus their mean were used to evaluate the limits of agreement between sessions [39].

Second, we used generalized estimating equations (GEE) to assess the longitudinal changes in blood glucose concentration, pFMD_v_ and mFMD_v_ indices, blood flow velocity, %FMD, and diameter with glucose loading. GEE are best suited to our study design and longitudinal data while they take into account the correlation between repeated measurements within individuals over time [40]. GEE models thus included session, time and interaction terms between session and time. The latter enabled to additionally investigate whether both sessions showed similar results at corresponding time-points [40]. Time (treated as a dummy variable with the fasting time-points as reference categories) was considered the independent variable and the repeated measurements and derived indices as dependent variables. In the analyses an exchangeable correlation structure was used [40].

ICCs were obtained with STATA software package version 9.2 (STATA Corp., Texas, USA) and GEE were analyzed with SPSS (Statistical Package for Social Sciences, version 20, IBM Corp., USA). A two-sided p-value <0.05 was considered statistically significant.

## Results

The 22 participants were 23.5 ± 5.8 years of age (mean ± SD), had a normal body mass index (23.2 ± 3.2 kg/m^2^), and were normotensive (systolic blood pressure 119 ± 8 mmHg; diastolic blood pressure 67 ± 4 mmHg), normoglycemic (fasting glucose 4.5 ± 0.3 mmol/l) and non-smokers (none n = 20, occasional n = 2, daily n = 0). Blood glucose concentrations, brachial artery diameter and blood flow velocity derived parameters, %FMD and both FMD_v_ indices are presented for each time-point and for each session in Table 1. None of the variables did significantly differ between sessions at any corresponding time-point (all p-values ≥0.071).

**Table 1:** Blood glucose and brachial artery flow-mediated dilation parameters during session 1 and 2, and intra-class correlations.

Parameter	Session 1						Session 2						ICC	
	t = - 40	t = - 15 / t = 0	t = 30	t = 60	t = 75	t = 120	t = - 40	t = -15 / t = 0	t = 30	t = 60	t = 75	t = 120	Intra- session	Inter-session
Glucose, mmol/l	4.5±0.4	4.4±0.3	6.9±1.2	6.5±1.9	6.1±1.7	4.8±1.3	4.5±0.3	4.6±0.3	6.6±1.0	6.1±1.6	5.9±1.3	4.5±1.1	-	-
diameter														
D_base_, mm	3.90±0.44	3.91±0.46	3.91±0.46	-	3.93±0.48	-	3.92±0.56	3.92±0.51	3.87±0.54	-	3.86±0.38	-	0.960	0.919
∆D_peak_, mm	0.22±0.12	0.22±0.10	0.19±0.09	-	0.21±0.13	-	0.22±0.10	0.23±0.10	0.20±0.12	-	0.22±0.13	-	0.675	0.550
∆D_peak_/D_base_, %	5.82±2.96	5.65±2.70	4.89±2.52	-	5.60±3.55	-	5.76±2.67	5.99±2.55	5.24±3.47	-	5.74±3.25	-	0.677	0.526
flow velocity														
v_base_, pxl	12±5	10±5	7±4	-	8±4	-	13±6	10±5	6±3	-	6±3	-	0.669	0.650
∆v_peak_, pxl	44±13	44±12	42±14	-	50±16	-	48±14	46±13	44±15	-	47±16	-	0.617	0.530
∆v_mean_, pxl	10±4	10±4	8±3	-	11±4	-	11±3	10±3	9±4	-	10±4	-	0.374	0.341
∆v_peak_/v_base_, %	410±144	493±157	790±422	-	766±297	-	453±241	536±235	881±368	-	868±408	-	0.536	0.521
∆v_mean_/v_base_, %	94±33	114±47	160±86	-	171±069	-	103±56	117±59	184±077	-	190±088	-	0.391	0.416
FMD_v_ indices														
(∆D_peak_/D_base_) /(∆v_peak_/v_base_)	0.016±0.012	0.012±0.006	0.008±0.006	-	0.009±0.007	-	0.017±0.013	0.014±0.010	0.008±0.007	-	0.008±0.007	-	0.589	0.565
(∆D_peak_/D_base_)/ (∆v_mean_/v_base_)	0.079±0.060	0.057±0.034	0.039±0.025	-	0.037±0.026	-	0.076±0.058	0.068±0.051	0.035±0.030	-	0.038±0.033	-	0.521	0.530

Data are means ± SD and intra-class correlation coefficients (ICC); t, time moment in minutes, with t = - 40, t = -15 (for brachial flow-mediated dilation measurement) and t = 0 (for glucose) as time-points before glucose intake (i.e. fasting time-points) and t = 30, t = 60, t = 75 and t = 120 as time-points after glucose intake; ICC calculated over the fasting time-points by ANOVA; n = 22. D_base_, baseline diameter; ∆D_peak_, peak change in diameter; ∆D_peak_/D_base_ is the %FMD measure; v_base_, baseline flow velocity; ∆v_peak_, peak change in flow velocity; ∆v_mean_, mean change in flow velocity; ∆v_peak_/v_base_, peak change in flow velocity relative to its baseline flow velocity; ∆v_mean_/v_base_, mean change in flow velocity relative to its baseline flow velocity; ∆D_peak_/D_base_ divided by ∆v_peak_/v_base_ is pFMD_v_; ∆D_peak_/D_base_ divided by ∆v_mean_/v_base_ is mFMD_v_; pxl, pixels.

### Reproducibility

The intra- and inter-session CVs for pFMD_v_ were 43 and 45% and for mFMD_v_ 51 and 51%, respectively. Those for the baseline blood flow velocity were 27 and 28%, those for the relative peak change in blood flow velocity 28 and 29%, those for the relative mean change in blood flow velocity 38 and 36%, those for the relative peak change in diameter (%FMD) 23 and 32%, and those for the baseline diameter 2.4 and 3.5%, respectively.

Inter-session ICCs for the FMD_v_ indices were comparable with those of %FMD (within 0.526–0.565), despite a somewhat lower ICC for the Δv_mean_/v_base_ component of the mFMD_v_ (Table 1). Intra-session ICCs were similar for the pFMD_v_ and mFMD_v_ indices and for the velocity parameters (Table 1), but somewhat higher for the diameter parameters and %FMD. Bland-Altman plots indicated no dependency of differences between inter-session data with their mean for any of the flow-mediated dilation parameters (data not shown). Inter-session bias was very low (<7%) but variability was substantial: mean difference [limits of agreement] for pFMD_v_ was 0.001 [-0.013; 0.015] (mean value of 0.015), for mFMD_v_ 0.002 [-0.067; 0.071] (mean value of 0.070), and for %FMD 0.02% [-4.68%; 4.72%] (mean value of 5.8%).

### Blood glucose

Blood glucose concentration rose from 4.5 mmol/l at baseline (no difference between -40 min and 0 min; p = 0.88) to 6.8 mmol/l at 30 min (p<0.001) after glucose intake, and gradually returned to 4.6 mmol/l at 120 min (p<0.001, compared to 30 min level; Table 1). Brachial mean arterial pressure did not change with glucose loading (p>0.23).

### Fasting values and changes in pFMD_v_ and mFMD_v_ with glucose loading

**Figure 3:**
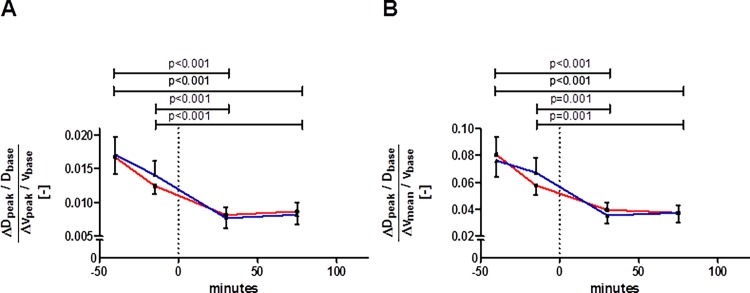
Changes in mFMD_v_ (A) and in pFMD_v_ (B) with glucose loading. Glucose was ingested at time-point 0 minutes (dashed vertical line). Red line: session 1, blue line: session 2; whiskers indicate standard error. Data did not differ between sessions at any corresponding time-point (all p-values ≥ 0.176, n = 22). The p-values on top of the graphs refer to differences between pre and post glucose intake (of the mean of both sessions). Abbreviations as defined in Table 1.

Both pFMD_v_ and mFMD_v_ indices showed fasting values well below 1 (Table 1 and Fig. 3). Compared to fasting (either at -40 min or at -15 min), both pFMD_v_ and mFMD_v_ were significantly (p≤0.001, Fig. 3A and 3B) and consistently reduced after glucose intake (about 45% lower; Table 2).

**Table 2:** Tracking performance of FMD_v_ compared to existing FMD indices.

FMD index	Calculation (from present study data)	Reference	Reduction	p-value
ΔD_peak_	= D_peak_–D_base_	-	11%	≥0.048
%FMD	= (D_peak_–D_base_)/D_base_*100	Celermajer et al.[35]	11%	≥0.074
pFMD_v_	= (D_peak_–D_base_)/D_base_/((v_peak_–v_base_)/v_base_)	-	47%	<0.001
mFMD_v_	= (D_peak_–D_base_)/D_base_/((v_mean_–v_base_)/v_base_)	-	44%	≤0.001
D_peak_/D_base_	idem	Atkinson et al.[34]	11%	≥0.074
FMD/shear_AUC_peak_	= (D_peak_–D_base_)/D_base_/Δv_AUC_peak_	Pyke et al.[9]	20%	≥0.016
FMD/shear_AUC_	= (D_peak_–D_base_)/D_base_/Δv_AUC_	Pyke et al.[9] Padilla et al.[36]	38%	≤0.038
ΔD_peak_/Δv_peak_	= (D_peak_–D_base_)/(v_peak_–v_base_)	-	3%	≥0.639
ΔD_peak_/Δv_mean_	= (D_peak_–D_base_)/(v_mean_–v_base_)	-	3%	≥0.710
FMD/Δv_peak_	= (D_peak_–D_base_)/D_base_/(v_peak_–v_base_)	-	4%	≥0.811
FMD/Δv_mean_	= (D_peak_–D_base_)/D_base_/(v_mean_–v_base_)	-	0%	≥0.712

Diameter and velocity parameters as defined in the text. Reduction percentages indicate the maximal reduction found after glucose intake (mostly 30 min after). Note that for the two indices normalizing for area under the curve (AUC) taking into account viscosity is not relevant in this evaluation of relative reductions with glucose; hence the calculations as specified are appropriate.

### Change in blood flow velocity with glucose loading

**Figure 4:**
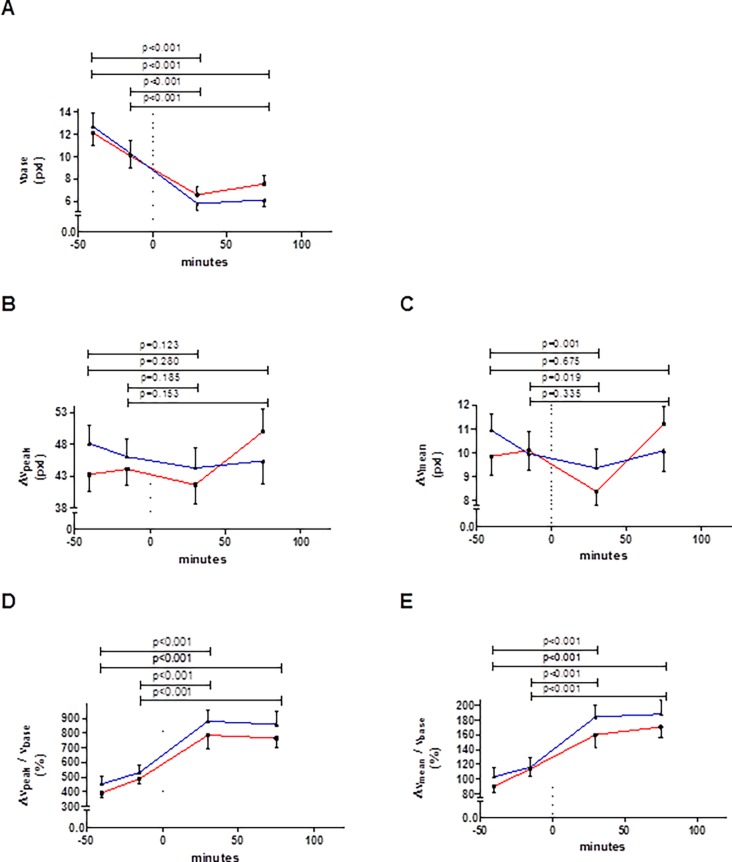
Changes in the baseline blood flow velocity (A), and the absolute changes in peak (B) and mean (C) blood flow velocity and relative changes in peak (D) and mean (E) blood flow velocity with glucose loading, as observed during flow-mediated dilation assessments at the 4 time-points indicated. Glucose was ingested at time-point 0 minutes (dashed vertical line). Red line: session 1, blue line: session 2; whiskers indicate standard error. Data did not differ between sessions at any corresponding time-point (all p-values ≥ 0.071, n = 22). The p-values on top of the graphs refer to differences between pre and post glucose intake (of the mean of both sessions). Abbreviations as defined in Table 1.

Compared to fasting, baseline blood flow velocity was consistently lower after glucose intake (p<0.001, Fig. 4A). The absolute *peak* hyperemic change in blood flow velocity did not change after glucose intake (p≥0.123, Fig. 4B). The absolute *mean* change in blood flow velocity showed a similar pattern, but was significantly lower at +30 min (p≤0.019, Fig. 4C). Compared to fasting, both Δv_peak_/v_base_ and Δv_mean_/v_base_ were significantly (p<0.001) and consistently higher after glucose intake (Fig. 4D and 4E).

### Change in %FMD with glucose loading

**Figure 5:**
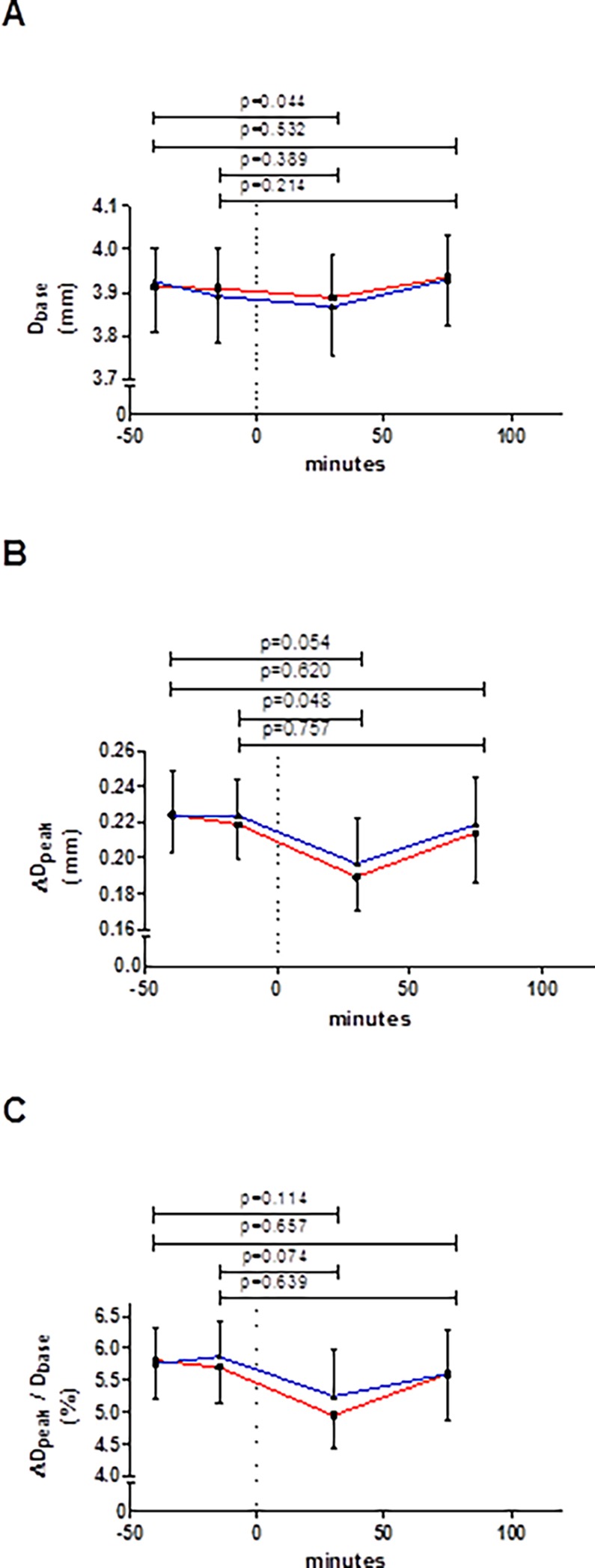
Changes in (A) baseline diameter and (B) the absolute and (C) the relative (i.e. %FMD) peak changes in brachial artery diameter. Glucose was ingested at time-point 0 minutes (dashed vertical line). Red line represents session 1 and blue line session 2; whiskers indicate standard error. Data did not differ between sessions at any corresponding time-point (all p-values ≥ 0.568, n = 22). The p-values on top of the graphs refer to differences between pre and post glucose intake (of the mean of both sessions). Abbreviations as defined in Table 1.

The baseline diameter of each flow-mediated dilation assessment remained constant throughout most of the experiment (Fig. 5A), but did show a significant decrease 30 min after glucose intake compared to the first fasting measurement at -40 min (p = 0.044). Compared to fasting (either at -40 min or at -15 min), at 30 min after glucose intake the absolute peak change in diameter was decreased, but it was near normal again after 75 min (Fig. 5B). Similarly, %FMD tended to be reduced 30 min after glucose intake (about 11% lower; Table 2), but did not differ from the fasting value at 75 min post glucose (Fig. 5C).

### Additional analyses

None of the parameters (Table 1) differed between the repeated fasting assessments (all p-values ≥0.31) except for v_base_ (p = 0.001), for Δv_peak_/v_base_ (p = 0.006), for pFMD_v_ (p = 0.013), and borderline significantly for Δv_mean_/v_base_ (p = 0.066) and mFMD_v_ (p = 0.078).

To ascertain whether the above results for the FMD_v_ indices are not merely explained by their components, we additionally evaluated the following absolute response-stimulus ratios ΔD_peak_/Δv_peak_ and ΔD_peak_/Δv_mean_, and %FMD/100 normalized to peak and mean hyperemic blood flow velocity increase: (ΔD_peak_/D_base_)/Δv_peak_ and (ΔD_peak_/D_base_)/Δv_mean_. These indices showed average reductions of less than 5% after glucose intake and none of these were statistically significant (all p-values ≥0.6; Table 2).

The indices normalizing the dilatory response to the area under the curve (AUC) of shear rate (reflected by relative changes of blood flow velocity with glucose; on top of baseline) showed greater reductions than %FMD and D_peak_/D_base_, but smaller reductions than both FMD_v_ indices (Table 2). AUC integration till peak velocity time (as for FMD/shear_AUC_peak_) appeared proportionally effective in capturing changes in stimulus with glucose, while integration over the full hyperemic period (as for FMD/shear_AUC_) enabled better detection of changes in local brachial artery flow mediated dilation (Table 2).

Times to peak hyperemic velocity or to peak dilatory response did not change with glucose (all p-values >0.1). The mean difference between the velocity and diameter peak times was 39±23 seconds, clearly showing an integrator-like response from the system (as illustrated by the measurement example used in Fig 1.)

## Discussion

The present study shows that proposed (shear stress normalization) performance indices have similar reproducibility, also when compared to %FMD. Both pFMD_v_ and mFMD_v_ indices (capturing the stimulus-response relation) showed more clearly a reduction after glucose loading than %FMD and related indices (capturing the response only). Consideration of either the peak or the mean hyperemic change in blood flow velocity in FMD_v_ did not lead to obvious differences in performance. These findings suggest a control systems approach to the quantification of shear stress normalization may improve detection of changes in brachial artery flow-mediated dilation in acute intervention studies. However, the interpretation of our findings require further discussion.

### Reproducibility and reliability

The present data corroborates the considerable measurement variability of %FMD, even under well-controlled conditions. Since the reproducibility of the FMD_v_ indices and %FMD proved similar, we need to conclude that taking into account the full stimulus-response relation (instead of the response only) does not necessarily improve reproducibility. However, our approach does improve the detection of changes with glucose loading (Table 2). This becomes evident when effect size is considered in relation to measurement variability. The change in the FMD_v_ indices after glucose intake compared to baseline, normalized to their intra-session CVs, was about 1 (for pFMD_v_ 47%/43% and for mFMD_v_ 44%/51%), while for %FMD the ratio was only 0.52 (11%/23%).

Agreement between sessions in terms of bias was good, with relative mean differences below 7% for the FMD_v_ indices and %FMD. For %FMD this compares favorably with previously reported relative mean differences of 25% (1.6/6.5*100%) [14] and 17% (1.1/6.5*100%) [18]. The lower value may be explained by the fact that we considered the average of two baseline flow-mediated dilation assessments per session. Furthermore, the observed 23% and 32% of intra- and inter-session CVs for %FMD fall within the previously reported range of 13–50% [14–18].

### Summary and interpretation of measurements

**Figure 6:**
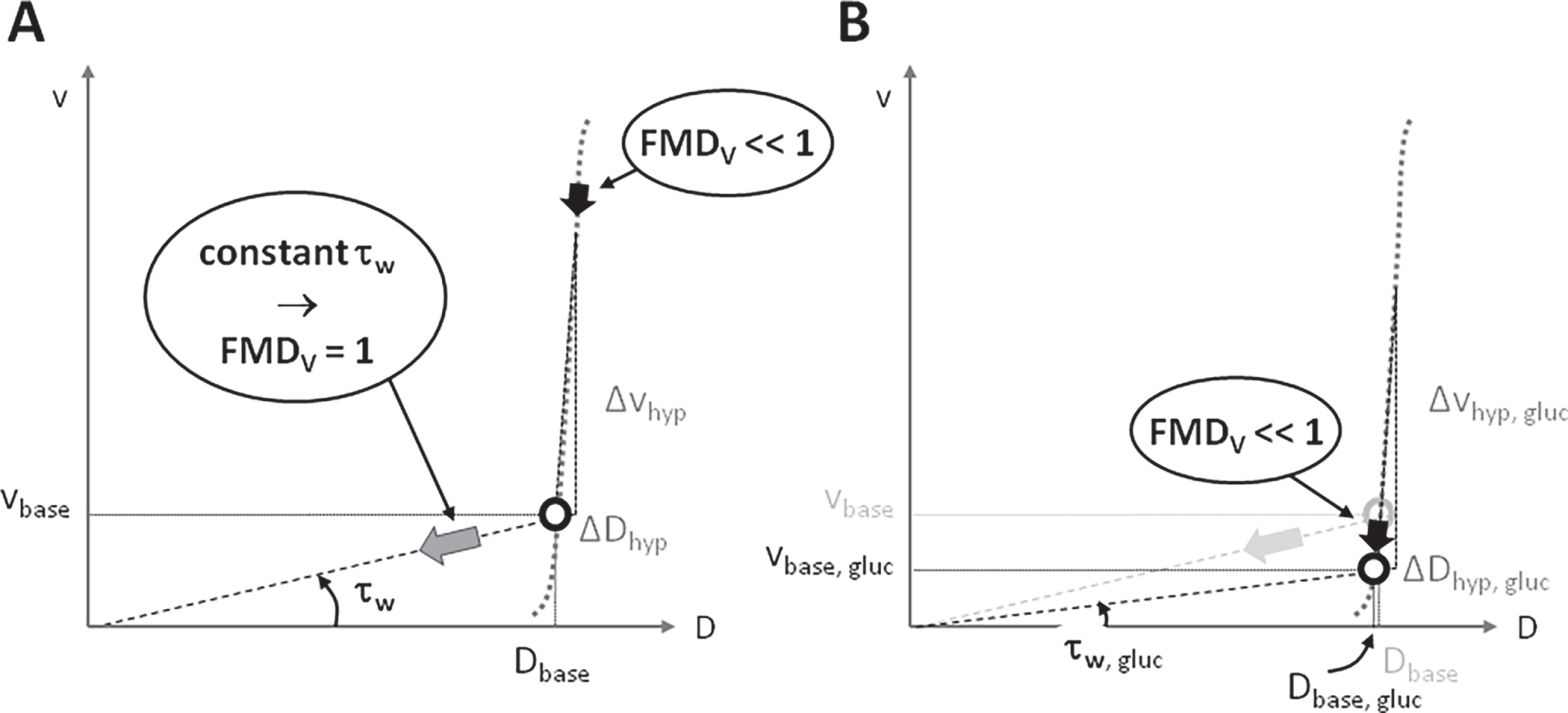
Summary and interpretation of measured changes in local brachial artery flow velocity (v) and diameter (D) after glucose loading. Please note that scales are all conform the relative magnitudes of the effects observed in the study group. Panel A shows the steady-state working point (circle) and hyperemic changes (Δ) under the fasting condition. The hyperemic changes are not aligned with the ideal response where wall shear stress (_w_ ~ v/D) would be kept constant perfectly, as indicated by the grey block arrow. FMD_V_ would be equal to 1 in that case. The poor shear stress normalization performance (FMD_V_ << 1; black block arrow) under fasting conditions is corroborated by the shift in the steady-state working point with glucose loading shown in panel B (black block arrow). This analysis indicates that wall shear stress normalization under normal (fasting) conditions is already far from ideal, which does not materially change with glucose loading.

The simultaneous and co-localized measurements of blood flow velocity (v) and vessel diameter (D) by duplex ultrasound are summarized in Fig. 6. The diagrams depict the proportionate steady-states and hyperemic changes in v and D in the (v,D)-plane, for fasting and hyperglycemic conditions. Given a steady-state v and D working point, full normalization of local wall shear stress by vessel dilation would be achieved if v and D would change proportionately (grey block arrow, Fig. 6A), in which case [Δv_hyperemic_/v_steady-state_] would equal [ΔD_hyperemic_/D_steady-state_]. Our data indicate shear stress normalization is far from ideal, as pFMD_v_ and mFMD_v_ are both well below 1 (Fig. 6A). Fig. 6B depicts the disproportionate shift in steady-state v and D we found with glucose loading. Notably, this shift is in line with the non-ideal response found with hyperemia and is responsible for the clear reductions in pFMD_v_ and mFMD_v_ with glucose loading (Table 2).

### Accounting for the stimulus-response relationship in FMD assessment

First, it should be emphasized that accounting for the full stimulus-response relationship as proposed has no intention of improving classification of subjects [31,34,41], but is the direct consequence of considering the continuous *physical* interrelationship between local wall shear stress, vessel diameter and blood flow velocity (Eqs. 1–3). It has been suggested that statistical adjustments could be more appropriate for subject classification [31,34,41]. However, for tracking changes *within* individuals stimulus-response relation should be respected and, hence, implies considering relative changes in flow velocity and diameter in relation (cf. Introduction). It should be noted that the assumptions underlying our approach includes are identical to existing and well-accepted approaches in the field [3,6,9,29,31,33,35,36].

As put forward in the introduction, local blood flow velocity and diameter are continuous opposing influences that modulate wall shear stress (τ_w_ ~ v/D; Eq.1). The present data indicate that when only the dilatory response is considered, i.e. by using %FMD [35] or D_peak_/D_base_ [34] to quantify the effective action of brachial artery flow-mediated dilation in response to (distal) hyperemia, the reduction with glucose is underestimated (11%) and harder to detect (Table 2). By capturing the *stimulus*-response relationship, the proposed FMD_v_ indices more robustly reflect changes with glucose loading (Table 2), although the ability of the brachial artery to locally normalize wall shear stress by dilation appears poor. The AUC-normalized indices we calculated also showed improved detection, as expected from previous work of others [9,36], although less convincing.

We investigated two possible parameters to capture the flow stimulus: the relative *peak* blood flow velocity and the relative *mean* blood flow velocity. It is generally agreed that the time integral (i.e. the mean blood flow velocity) should be considered to quantify the flow-stimulus responsible for dilatory response [9]. The problem with this approach is to define a proper time window, because so far little has been elucidated about the time constants/delays of the various elements acting with flow-mediated dilation (mechano-sensing, signal transduction, phosphorylation, NO diffusion, muscle relaxation). Even if the time integral is properly restricted (e.g. by visual inspection), it does not provide a conclusive quantification of the effective flow-stimulus because of its transient nature. For a short stimulus, the peak of the flow-stimulus may probably be more relevant than the mean flow-stimulus via the time integral, but we did not find differences between both FMD_v_ indices. In contrast, for the AUC-normalized indices the integrating nature of the system is apparent from the improvement in tracking ability when integrating beyond peak-velocity time (Table 2). The work of Padilla et al. and Pyke et al. shows that integration beyond 1–2 minutes practically covers the full AUC of blood velocity [9,36]. Our 180-second integration includes the full AUC, but does not presume a specific temporal relationship between stimulus and response, as done by some [9].

### Justification of glucose challenge as model intervention

In the present study, we utilized an oral glucose challenge to comparatively evaluate our new approach to existing approaches. It is important to first note that previous reports lack consistency. Some studies (in healthy individuals as well) reported relative reductions in %FMD with glucose intake of ~28% after 30 minutes [19], of ~18% [20] and of ~65% [21] after 1 hour, and of ~43% after 2 hours [22], all of which were statistically significant. Other studies indicated relative reductions of 11% [23] and of 44% [24] after 1 hour, which were not significant (like ours; 11% relative reduction %FMD after 30 minutes, Fig. 3 and Table 2). Some studies did not observe a change or observed an increase with glucose intake [10,26]. Our findings on %FMD with glucose intake therefore appear in line with literature, though one should remain aware of the various methodological factors, e.g. sample size.

### Strengths and limitations of the study

Strengths of the present study include:

- Flow-mediated dilation measurements were executed according to the latest international recommendations [4,6–8].
- Extraction of the diameter and blood flow velocity parameters was semi-automatic and fully blinded.
- The continuous assessment over 13 minutes [8]: (i) provided detailed blood flow velocity and diameter curves, excluding time ambiguity with respect to the derived blood flow velocity and diameter variables, and (ii) enabled visual inspection of the blood flow velocity and diameter curves for artifacts and/or drift, allowing verification of the automatically determined values.
- The repeated execution of the protocol enables comprehensive assessment of reproducibility, which contributed to the reliability of our observations.

A number of issues limit the scope of our study and conclusions.

Our results were obtained in healthy men, precluding generalization to women, smokers, obese and diabetic subjects. As such, our study did also not address differences in glucose metabolism and related macro- and microvascular effects on our approach. Future studies are hence necessary to establish the (clinical) value of our quantitative approach, with or without the use of an OGTT challenge as part of the assessment. As no gold standard method exists for benchmarking our FMD_v_ indices, further studies should focus on improved discrimination of the micro- and macrovascular effects governing flow-mediated dilation in vivo.

The FMD_v_ indices are based on a steady state relationship between flow-stimulus and dilation response, which is not necessarily suited to transient hyperemic flow-stimuli [7,29]. Although not statistically significant, we did note an apparent disagreement between sessions for both the +75 min peak and mean hyperemic changes in flow-velocity. A stepwise review of our data revealed that the difference as evident in Fig. 4B and C result from considerable intra-individual variability in some subjects. This chance variability seems in line with the rather poor ICCs we found (Table 1). In practice, such variability might result from short-term physiological variability or slight out of plane motion of the vessel during deflation of the cuff, though the latter was not obvious from our recordings.

Within our study, the decrease in absolute baseline flow-velocity after glucose intake was an unexpected or at least a less intuitive finding. Given that mean blood pressure and brachial artery diameter did not change significantly, the decrease in flow-velocity should stem from an increase in peripheral resistance, as a direct effect of glucose on the distal microcirculation [42]. Unfortunately, our study did not include distal microcirculatory measurements, which limits further disentangling of the increased (relative) flow stimulus we observed after glucose intake.

The present analysis cannot exclude a carry-over effect between first and second flow-mediated dilation assessments (spaced 10 min apart in each session) potentially caused by repeatedly engaging the flow-mediated dilation apparatus. Despite our adherence to current guidelines, we cannot exclude that further acclimatization of subjects or repeated hyperemic challenges as such affected our measurements.

## Conclusions

We conclude that, from a control systems perspective, wall shear stress normalization at brachial artery level is normally far from ideal (FMD_v_ << 1). This poor performance of the flow-mediated dilation system seems not to change materially after glucose loading. Nonetheless, the FMD_v_ approach improves detection of the effect of such intervention. The value of our approach in pharmacological or dietary intervention studies for detecting intrinsic changes in shear stress normalization in conduit arteries remains to be established.
